# Synchronous double cancers of primary hepatocellular carcinoma and intrahepatic cholangiocarcinoma: a case report and review of the literature

**DOI:** 10.1186/1477-7819-12-337

**Published:** 2014-11-10

**Authors:** Chao Wu, Dou-Sheng Bai, Guo-Qing Jiang, Sheng-Jie Jin

**Affiliations:** Department of Hepatobiliary and Pancreatic Surgery, Clinical Medical College of Yangzhou University, 98 West Nantong Road, Yangzhou, Jiangsu 225001 P.R. China

**Keywords:** Liver, Double hepatic cancer, Hepatectomy

## Abstract

We report a case of double primary liver cancer comprising hepatocellular carcinoma (HCC) and intrahepatic cholangiocarcinoma (ICC). A 58-year-old Chinese man without obvious liver cirrhosis was diagnosed with multiple HCC in segment V (SV) and segment VIII (SVIII) of the liver. Preoperative abdominal magnetic resonance imaging revealed two solid masses in SV and SVIII. We performed hepatic resection of both segments. The tumors in SV and SVIII were pathologically diagnosed as HCC and ICC, respectively. Immunohistochemically, the HCC in SV was positive for carcinoembryonic antigen and negative for α-fetoprotein (AFP) and cytokeratin (CK), while the ICC in SVIII was negative for both AFP and CK. These observations confirmed the diagnosis of double primary liver cancer (HCC and ICC). Double primary liver cancer is extremely rare. We herein review previous reports of patients with a histological diagnosis of double primary liver cancer. Based on the findings of this case and the literature review, we speculate that the imaging findings of double primary hepatic cancer conform to the pathologic findings.

## Background

Hepatocellular carcinoma (HCC) and intrahepatic cholangiocarcinoma (ICC) are the two main primary liver cancers. The occurrence of synchronous double cancers comprising different primary hepatic tumors is very low. Only 33 cases of ICC with HCC have been reported [1], and a survey of previous reports produced only 18 cases of double primary liver cancers that were synchronously resected [2]. We herein report a patient who underwent hepatectomy for double primary liver cancer (HCC and ICC) without a background of obvious liver cirrhosis.

## Case presentation

**Figure 1:**
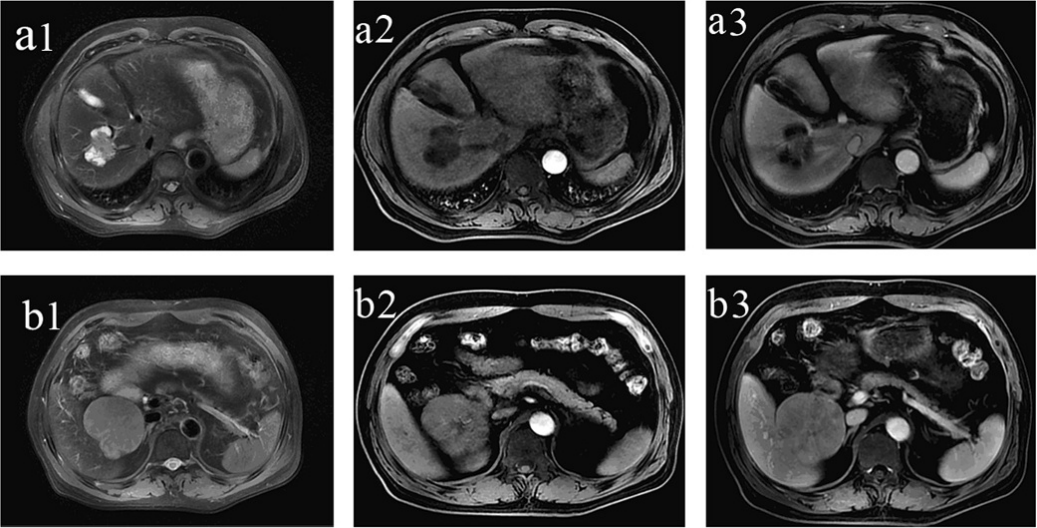
**Abdominal magnetic resonance imaging depicted two isolated tumors.** One measured 4.0 cm in diameter and was present in the left segment of the liver (SVIII, **a1**). The other measured 6 cm in diameter and was present in the inferior superior segment of the liver (SV, **b1**). The larger tumor showed enhancement **(b2)**, and the smaller tumor showed contrast enhancement **(a2)** in the center of the tumor in the hepatic arterial phase. In the portal phase, the larger tumor became less dense than the liver parenchyma **(b3)**, but the centrality of the smaller tumor showed continuous enhancement **(a3)**.

**Figure 2:**
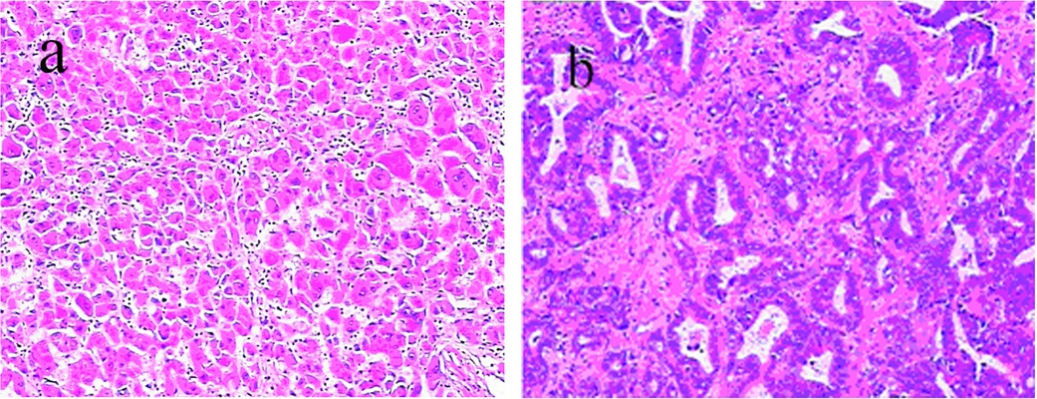
**Light microscopy showing the two tumors (hematoxylin-eosin staining, ×100).** The hepatocellular carcinoma comprised polygonal cells in a trabecular pattern **(a)**, and the cholangiocarcinoma contained mucin-producing glands **(b)**.

A 58-year-old man presented with a poor appetite and abdominal tenderness in the area of the liver. He was subsequently admitted to Northern Jiangsu People’s Hospital in June 2013 for further examination of a hepatic nodule detected on thoracic computed tomography. His medical history included cerebral infarction and hypertension, but he had no history of exposure to any hepatotoxic chemicals or blood transfusions. Physical examination findings upon admission were normal with the exception of slight abdominal tenderness in the area of the liver. Laboratory examination produced the following results: red blood cell count, 3.22 × 10^12^/l; hemoglobin, 95 g/l; white blood cell count, 12.7 × 10^9^/l; albumin, 40.1 g/l; globulin, 18.4 g/l; total bilirubin, 13.2 μmol/l; and prothrombin time, 13.8 s. The alanine aminotransferase, aspartate aminotransferase, and lactate dehydrogenase levels were within their normal ranges. Hepatitis B virus-related antigen and antibody were negative with the exception of hepatitis B surface antibody (578.200 IU/l) and hepatitis B core antibody. The carbohydrate antigen 19-9, carbohydrate antigen 242, α-fetoprotein, and carcinoembryonic antigen levels were 331 U/ml, 167.31 U/ml, 9.53 ng/ml, and 3.61 ng/ml, respectively. Abdominal magnetic resonance imaging identified two liver tumors: one in segment V (SV, 6 cm in diameter) and one in segment VIII (SVIII, 4 cm in diameter) of the right lobe (Figure 1). The patient underwent two systemic partial liver resections and cholecystectomy. The parenchymal margins were uninvolved by both tumors. Histopathologic examination of the liver specimens revealed two types of tumors with two different histological patterns. The hepatocellular component of SV exhibited trabecular structures with large polygonal cells, which were immunohistochemically positive for carcinoembryonic antigen (Figure 2a). The cholangiocellular population of SVIII comprised glandular structures with intraluminal mucin and a diffuse distribution (Figure 2b). The background liver parenchyma was noncirrhotic. The patient’s postoperative course was uneventful, and he was discharged on postoperative day 15. He was alive without recurrence at the time of this report. Written informed consent was obtained from the patient.

## Discussion

Combined HCC and cholangiocarcinoma (cHCC-CC) is a rare primary liver tumor containing unequivocal elements of both HCC and ICC. In 1949, Allen and Lisa [3] first classified cHCC-CC into three groups: type A (HCC and ICC present at different sites within the same liver), type B (HCC and ICC present at adjacent sites and mingling with each other but still recognizable as distinct tumors), and type C (HCC and ICC components combined within the same tumor and indistinguishable as separate entities). Most cHCC-CCs exhibit an HCC component accompanied by cholangiocarcinoma as a collision tumor. The reported incidence of cHCC-CC in one study was only 0.54% among 18,843 pathologically diagnosed primary liver cancers in Japan [4]. Moreover, the frequency of synchronous double primary cancers in the liver is much lower than the frequencies of combined and mixed types [3]. The cellular origin of cHCC-CC is still unclear. Several studies have shown that activated hepatic progenitor cells are considered to be involved in carcinogenesis, and at least some cHCC-CCs are suggested to arise from hepatic progenitor cells [5]. Hepatic progenitor cells have the potential to differentiate into either hepatocytes or cholangiocytes depending on the damaged cell population. However, this theory can only explain the growth pattern of types A and B described above. Therefore, we speculate that the two masses composing the double primary hepatic cancer herein reported arose respectively.

Double primary hepatic cancer is an unusual hepatic tumor comprising two histologically distinct tumors that exist simultaneously within the liver. The imaging findings of HCC often exhibit an area of hyperenhancement in the early phase and hypoenhancement due to washout of contrast medium in the late phase; otherwise, the ICC shows peripheral enhancement in the early and late phases. In the present patient, because both HCC and ICC occurred in the liver simultaneously, the imaging findings of these two distinct tumors could be compared in the same condition. In our patient, slight contrast enhancement was observed at the centrality of the mass in SVIII on magnetic resonance imaging obtained during the hepatic arterial and portal venous phases; concentric filling was shown on the delayed images (Figure 1a). In contrast, the other mass in SV was highly enhanced and its density gradually decreased in the portal venous and delayed phases (Figure 1b). We also reviewed four additional studies of double primary liver cancer (HCC and ICC), and the imaging findings were approximately the same as those in our patient [1, 6–8]. Therefore, we speculate that the imaging findings of double primary hepatic cancer conform to the pathologic findings.

Surgery remains the only treatment option offering a potential cure for localized disease, especially in noncirrhotic patients, although the risk of recurrence may be higher than that associated with pure HCC or ICC [9]. Lymph node spread is a well-known mode of dissemination in patients with ICC. Thus, it can by hypothesized that lymph node sampling and removal of positive nodes in patients with cHCC-CC is equally as important as in ICC. Portolani *et al*. [10] reviewed 18 patients with cHCC-CC who underwent surgical resection and reported that nodal metastasis was a negative prognostic value for both overall and disease-free survival, with the mean survival duration in node-positive patients being only 14.7 months compared with 38.5 months in node-negative patients. Surgery for HCC usually requires only resection of the liver tumor because of the low incidence of nodal metastases, while the presence of cholangiocarcinoma makes lymph node dissection essential. Wider use of intraoperative frozen biopsy, especially for lesions not typical for HCC, can be suggested for the diagnosis and optimal management of cHCC-CC.

Previous studies have reported a poorer prognosis for cHCC-CC than for either HCC or ICC. The prognosis of combined HCC and ICC has generally been recognized to be poorer than that of ordinary HCC because of the ability of the combined type to metastasize to many organs [11]. Cao *et al*. [12] reported the survival data of 14 patients who had cHCC-CC. The median overall survival (OS) was 18 months, and the 1-, 3-, and 5-year OS rates were 60.0%, 28.9%, and 23.1%, respectively. Multivariate analysis indicated that the ICC tumor size, presence of lymph mode metastasis, and histological differentiation of the ICC component were independent risk factors for OS.

## Conclusions

In summary, we have reported a case of double primary liver cancer (cHCC-CC) confirmed by pathological and immunohistochemical analyses. Although double primary liver cancer is rare, it must be considered as a differential diagnosis of liver tumors. Imaging findings may be helpful to achieve a correct preoperative diagnosis. If in doubt, intraoperative frozen section analysis may be necessary to select the correct surgical approach.

## Consent

Written informed consent was obtained from the patient for the publication of this case presentation and accompanying images. A copy of the written consent is available for review by the Editor-in-Chief of this journal.

## Abbreviations

AFP: α-fetoprotein
cHCC-CC: combined HCC and cholangiocarcinoma
CK: cytokeratin
HCC: hepatocellular carcinoma
ICC: intrahepatic cholangiocarcinoma
OS: overall survival
SV: segment V
SVIII: segment VIII.
